# Study of Empyema Cases at Camp Upton

**Published:** 1919-01

**Authors:** 


***A Study of Empyema Cases at Camp Upton.*** By Major HARLOW BROOKS, M. D., and Major RUSSELL L. CECIL, M. D. *The Archives of Internal Medicine,* September, 1918.

At Camp Upton a close study of pneumonia revealed unusual bacteriology, different symptoms and an altogether modified pathologic picture. It was also found that the usual operative procedures in empyema gave disastrous results.

Conjoint clinical and laboratory studies were resorted to, and demonstrated the presence of a specific and violent organism — the streptococcus haemolyticus.

The medical authorities feel quite certain that streptococcus empyema may develop from a primary streptococcus pneumonia.

In the pneumococcus empyema the patient may not be very ill, though there is rise in temperature. On exploring the chest, cloudy creamy pus is found, containing many pus cells and capsulated pneumococci.

In the fatal cases of this group the men all died early, apparently from the intensity and extent of the pneumonia rather than from the concomitant empyema.

The very early onset of streptococcus empyemas was demonstrated in hospital examinations on an average of four or five days after admission. It was seen that these empyemas are imposed on and do not merely succeed the active stage of pneumonia.

There have been so many errors regarding the diagnosis of empyema that it is well to record the symptoms usually met at the onset. In the streptococcus haemolyticus empyemas the sputum is rather characteristic; usually white or yellowish, viscid and mucopurulent.

Considerable febrile periods are succeeded by rapid oscillation, as in active sepsis. Pain in the side is a very inconstant symptom.

On percussion, moist crackling rales are nearly always to be heard. This empyema develops with surprising rapidity and often before a conclusive diagnosis of pneumonia, in addition to pleurisy, could be made.

Appearance of profuse sweat is one of the most constant signs. The purulent exudate is more pronounced than in ordinary pneumonia. Acceleration of the respiration is another suggestive sign, since when the pus begins to form there is a marked increase in the respiratory rate.

When exhaustion is particularly striking a definite diagnosis may be arrived at by applying the needle. The abdominal rigidity always looked for in this epidemic was strikingly absent in the eighty cases examined at Camp Upton. On the contrary a marked tenderness, very often deep, is used as a guide for needling.

Beyond question the single physical sign which doctors have come to consider as of most value in the detection of these effusions is alteration in percussion. A high pitched note or the flattening of the customary resonance is a sufficient indication for a tap. Pleuropericarditis is frequent and the very early appearance of this lesion is quite as remarkable as the appearance of the empyema itself. The bacteriology of the pericardial exudate coincides with that of the pleural fluid. Study of the total and differential white cell count has been very disappointing as a diagnostic measure.

The usual roentgen ray is advisable to indicate the extent and the nature of the pulmonary process, particularly stereoscopic plates, yet it should not be depended on entirely, since the needle in several instances showed fluid where the roentgen ray did not indicate it. A fine hypodermic needle from three to four cm. in length attached to an ordinary 5 cc. Luer syringe is best for the purpose. The Potain aspirator was used ordinarily at Camp Upton.

It has been observed that the bacteria are much more numerous in the streptococcic empyema than in the pneumococcic empyema. Cultures from the exudate show many small, elevated, opaque colonies of a pearl gray color, each surrounded by a sharply defined zone of hemolysis.

Observations made on streptococcus viridans empyemas, following pneumococcus pneumonias, showed that the majority of these cases were frank *lobar* pneumonia. The mortality in both groups was very high, being as much as 61 %.

Often the clinical picture suggests a phase of a general septicemia, but the rare occurrence of metastatic foci is definitely against this theory. It is probable that in the majority of cases streptococcus empyema develops from a broncho-pneumonia, the infectious organism being conveyed to the pleura either by the way of the lymphatics or by the direct extension of one of the small abcesses so frequently seen through the pleural coat.

The likelihood of infection from streptococci already in the mucous tract of patients before they contract the pneumonia seems to us a much more probable factor.

At necropsy, the fluid in some cases was frankly purulent, and in most cases the pleura was covered with a thick layer of fibrin which varied in thickness with the age of the empyema. It was closely adherent to the pleura. The lung was often atelectatic; on compression the bronchioles stand out markedly and are usually filled with pus, giving them the appearance of small tubercles or miliary abscesses. The pleural covering was also noticed to be converted into a thick coat of granulation tissue with a surface of fibrin and leukocytes.

In the pathologic conditions associated with empyema often a pocket of pus was found between the pericardial sac and the adjacent portion of the left lung. Small and multiple abcesses of the lung were the next most common pathologic finding. Bilateral empyema was rarely encountered.

For the treatment of the above mentioned infection it has been the experience of the physicians at Camp Upton not to use general anesthetics when operations are necessary. In pneumococcus empyema early operation is indicated, but such is not the case for streptococcus or sterile empyema. For the latter groups the operative measures should be resorted to when the pus becomes gelatinous and thick with fibrin and cells. As soon as the fluid is discovered (use of needle and roentgen ray), it is aspirated by the Potain aspirator if it proves to be embarrassing the respiration, otherwise not. Meantime the treatment of pneumonia is followed, digitalis is given freely in the early stages, and camphor, caffein, etc., are given as symptomatically indicated. When the patient coughs incessantly codein or morphin is used.

The usual attention must be given to elimination and to abundant aeration.

